# A Systematic Literature Review of Green and Sustainable Logistics: Bibliometric Analysis, Research Trend and Knowledge Taxonomy

**DOI:** 10.3390/ijerph17010261

**Published:** 2019-12-30

**Authors:** Rui Ren, Wanjie Hu, Jianjun Dong, Bo Sun, Yicun Chen, Zhilong Chen

**Affiliations:** 1 College of Defense Engineering, Army Engineering University of PLA, Nanjing 210042, China; renrui0801@163.com (R.R.); Chen-zl@vip.163.com (Z.C.); 2 College of Civil Engineering, Nanjing Tech University, Nanjing 211816, China; sunbo8079@163.com; 3 Research Institute for National Defense Engineering of Academy of Military Science PLA China, Beijing 100850, China; cyc-lgdx@foxmail.com

**Keywords:** sustainable logistics, freight transportation, green initiative, transport and environment, supply chain management, literature review, bibliometric, taxonomy

## Abstract

Ever-growing globalization and industrialization put forward impending requirements for green and sustainable logistics (G&SL). Over the past decades, G&SL initiatives triggered worldwide deliberations, aiming at easing negative transport externalities and improving supply chain performance. This review-based paper attempts to offer a joint quantitative and qualitative understanding for the overall evolutionary trend, knowledge structure, and literature gaps of the G&SL research field. Employing the science mapping approach, a total of 306 major paper published from 1999 to 2019 were retrieved, elaborated on, and synthesized. Visualized statistics regarding publication years, journal allocation/co-citation, inter-country/institution collaboration, influential articles, co-occurred keywords, and time view clusters of research themes were analyzed bibliographically. On this basis, a total of 50 sub-branches of G&SL knowledge were classified and thematically discussed based on five alignments, namely (i) social-environmental-economic research, (ii) planning, policy and management, (iii) application and practice, (iv) technology, and (v) operations research. Finally, the current knowledge obstacles and the future research opportunities were suggested. The findings contribute to portray a systematic intellectual prospect for the state quo, hotspots, and academic frontiers of G&SL research. Moreover, it provides researchers and practitioners with heuristic thoughts to govern transportation ecology and logistics service quality.

## 1. Introduction

Sustainable development has inspired many green and sustainable logistics (G&SL) activities to reduce the negative effects of freight transportation [1] and improve positive environmental and social feedbacks. From long-haul heavy-duty logistics to intra-city distribution, road-based freight transportation systems generate tremendous negative externalities in daily operations [2], including pollutant emissions, congestion, traffic accidents, noise, visual interference, infrastructure failure and resource waste [3]. Moreover, these negative externalities, together with the disadvantages of logistics system itself (e.g., limited intelligentization, personnel dependence and vulnerability [4]), further lead to the downgrade of supply chain performance at both enterprise level and regional level. With the rapid growth of logistics demand, the damage grows exponentially, which will eventually bring irreversible impacts to the economy and the whole ecosystem [5].

The operation management of physical distribution is one of the most significant and challenging sub-issues of the macro supply chain management (SCM) [6], because it involves real-time scheduling and coordination of hundreds of thousands of packages and containerized goods under a dynamic logistics scenario [7]. G&SL is defined as the planning, control, management, and implementation of logistics system through the advanced logistics technologies and environmental management, aiming to reduce pollutant emissions and improve logistics efficiency [8]. G&SL is not only concerned with providing customers with green products or services [9], but also with the green and sustainability of the entire lifecycle of the logistics process [10]. Various green logistics modes, activities, and behaviors were proposed and gradually realized from government rules to technological innovations. For example, the construction of green logistics network [11,12,13], reverse logistics [14], emission control [15], electric freight vehicle [16], modal shift and multimodal transportation [17], energy efficiency [18], collaboration [19,20], outsourcing [21,22], etc. A wide range of topics related to G&SL yielded substantial academic results and considerable practical performance. However, G&SL is still in its infancy and is far from meeting the challenges posed by the complexity of internal cooperation and uncertainties of external markets [1].

Previous studies reviewed G&SL from different perspectives. By reviewing 115 papers, Zhang et al. [10] analyzed the combinatorial optimization problems and swarm intelligence technique applied in improving G&SL performance. Qaiser et al. [23] conducted some brief statistics on the bibliometric information of 40 papers on G&SL. Bask and Rajahonka [8] mainly reviewed the role of environmental sustainability in multimodal freight transport decision-making. Based on 56 papers, Mangiaracina et al. [24] summarized the impact of business-to-customer transportation process on the environment. Arvidsson et al. [25] reviewed the sustainable measures for improving urban distribution efficiency. Pourhejazy and Kwon [26] conducted a survey on 380 articles published from 2005–2016 and revealed the application status of operations research technique in the supply chain optimization. The literature of green SCM was classified and reviewed by Srivastava [4] from a reverse logistics angle. This work was further enriched by Fahimnia et al. [27], who investigated the bibliographical information and trend of a majority of green SCM research through article co-citation network and keywords co-occurrence network.

However, based on the time of publication and the number of papers contained, the existing studies are outdated and incomplete, unable to provide a comprehensive analysis of the booming G&SL research in the past two years. Also, it is more difficult to integrate the multitudinous research directions to build a complete knowledge structure for G&SL. Therefore, it is of great theoretical and practical significance to objectively and quantitatively investigate the overall progress of G&SL.

This study aims to conduct a comprehensive review of the global G&SL literature, so as to explore the state-of-the-art, hotspots and research trend, as well as to build the G&SL knowledge classification system. Specifically, first, tracking and analyzing the evolution of the G&SL research field from (i) publication year and journals; (ii) countries, regions, and organizations; (iii) influential documents; (iv) keywords clustering and research themes. Second, establishing the knowledge taxonomy based on the scientometric results. Third, identifying the research gaps and the future research opportunities.

The novelty of this study lies in two aspects. One is to integrate the science mapping approach into the systematic literature review process to visualize the relationships among the G&SL literature. Science mapping approach is composed of data mining and bibliographic analysis, which can minimize subjective arbitrariness and grasp useful information to facilitate in-depth thematic analysis. Another is that this study further extends the bibliography to illuminate the emerging knowledge branches, gaps, and agendas in G&SL research, which will contribute to the improvement of G&SL practice and research innovation. The findings are expected to provide researchers and practitioners with a panoramic description and in-depth understanding of G&SL research. Additionally, the proposed knowledge structure can also be used as a handbook-like tool to further collect, analyze, and expand knowledge in the G&SL field and to provide references for other innovative logistics initiatives.

The rest is organized as follows. In Section 2, the outline of research method is introduced. Section 3 presents the results of the data collection and the results of five parts of scientometric analysis. Section 4 proposes the taxonomy of G&SL research based on the keywords clustering and discusses the knowledge branches in detail. The current research gaps and agenda are also identified. Section 5 summarizes the major findings and limitations.

## 2. Research Method

### 2.1. Overview of Review Protocol

This review-based study conducted a systematic investigation on the academic development of global G&SL research with the aids of science mapping. Science mapping is a quantitative analysis approach that uses mathematical statistics and visualization techniques to study bibliographic networks (e.g., academics, institutions, themes, keywords, and journals) in a specific field [28]. This approach has been widely applied in many academic fields, such as sustainable transportation [29], environment science [30], city logistics [31] and waste management [32] and can directly synthesize salient findings from the existing knowledge system.

**Figure 1:**
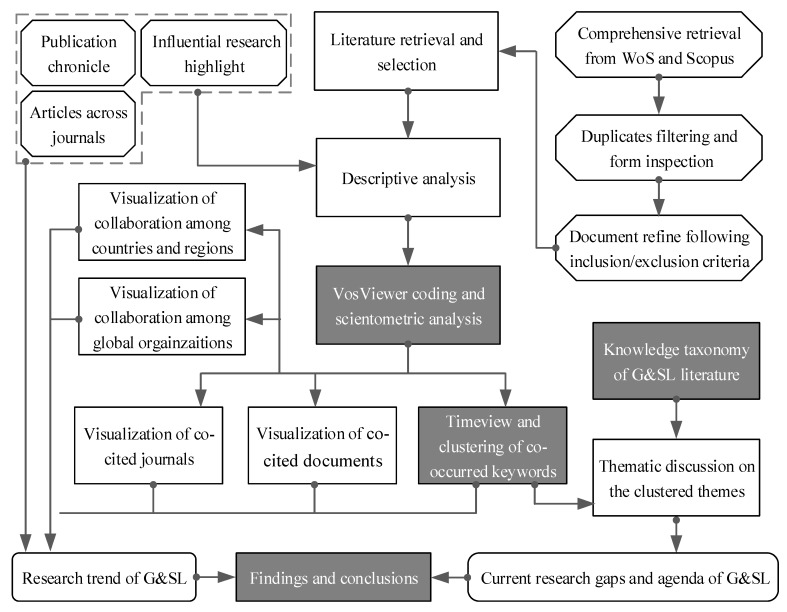
The flowchart of reviewing G&SL literature.

Figure 1 illustrates the detailed research process, consisting of three steps.

In step 1, the statistics was obtained after a comprehensive retrieval from two electronic databases, Web of Science (WoS) core and Scopus. Two rounds of selection were then performed to refine, classify, and encode the documents. The year publication trend, journal allocation and the most cited articles were described.

Four scientometric tests were carried out in step 2, namely (i) *Journal co-citation analysis*: to identify the most cited journals and the research domains they belong to. This analysis helps to reveal the distribution of published journals and cited journals of the reviewed documents, so as to identify popular journals in G&SL research domain. (ii) *Countries/organizations collaboration analysis*: to visualize the collaborative research network of G&SL among countries and organizations, so that the readers can quickly understand the partnerships between major research communities and institutions around the world. (iii) *Document co-citation analysis*: to highlight the influential G&SL articles and the corresponding reference relationships. By analysis of the papers with high citation, the emerging trend of scholars’ research interest to G&SL is easier to grasp. (iv) *Keywords co-occurrence analysis*: to map out the co-occurred time zone of the hotspots G&SL keywords and cluster them into several research themes. Network analysis of co-occurred keywords is used to clarify the knowledge structure of G&SL as well as to present the research hotspots and potential research opportunities in the future.

In step 3, the hierarchical knowledge structure of G&SL was proposed for thematic discussion.

The text mining software VOSviewer was adopted for science mapping, combining with another software CiteSpace to portray the time view of the clustered keywords based on the same data. VOSviewer, developed by van Eck and Waltman [33], is a comprehensive bibliometric analysis tool based on Visualization of Similarities (VOS) technology, which has unique advantages in clustering fragmented knowledge from different domains according to their similarity and relatedness. In the visualized networks, a node signifies a particular bibliographic item, such as organization, country, keyword or reference, etc. The node size represents the counting of the evaluated item namely citation or occurrence. Link denotes the co-citation, co-occurrence or collaboration relationship. The metric, total link strength (TLS), is outputted automatically by the software to reflect the correlation degree between any two nodes in the generated networks. A higher value of TLS, the higher importance and centrality of the item has [31]. Nodes with a high similarity were clustered together and distinguished by colors with other clusters, while the nodes with low similarity should be separated as far as possible. The similarity matrix can be calculated by Formula (1), where *c_ij_* is the co-occurred or co-cited times of item *i* and item *j*, *W_i_* and *W_j_* denote the node sizes of item *i* and item *j* respectively [33]. The stopping criterion of VOSviewer mapping is the minimal sum of weighted Euclidean distances of all items in each cluster [34], which can be expressed by Formula (2), where *x_i_* and *x_j_* are the positions of the nodes.

For a detailed operation manual of bibliographical experiments using science mapping approach, readers are advised to refer Jin et al. [28] and Hu et al. [31].

(1) $$S=\left[similarity_{ij}\right]=\left[c_{ij}\cdot W_{i}/W_{j}\right]$$

(2) $$E\left(X,S\right)=\sum_{i<j}similarity_{ij}{\left\|x_{i}-x_{j}\right\|}^{2},\;\sum_{i<j}\left\|x_{i}-x_{j}\right\|=1,\;\mathrm{for}\;\forall i,j$$

### 2.2. Literature Retrieval and Selection

The advanced retrieval function in Scopus and WoS core collection database was used to retrieve the G&SL related papers published during 1999 to August 2019 (see Table 1). To ensure the quality of the literature, the document types were restricted to research articles, while other types such as the conference proceeding, book chapter, letter or editorial material were excluded. The preliminary search yielded 1160 records. These records were imported into EndNote software for the first-round inspection to filter out duplicates and unqualified records in forms (e.g., article length and integrity). Additionally, those completely and partially irrelevant studies were removed. For example, an article entitled “Using logistics regression to analyze the sustainable procurement performance of large supply chain enterprises” was not the desired result. A total of 397 records were left after the first-round inspection. Then, the second-round selection was carried out by carefully reading the abstract of each document. The inclusion and exclusion criteria for this round focused on whether the document was consistent with the research topic, i.e., with green logistics initiatives, practices. and other G&SL innovations, rather than broader research, such as production, manufacturing or urban transportation. Unless it has a strong relation with G&SL. In particular, the following topics were excluded: (i) green design on the specialized logistics technology e.g., biomass and biofuel; (ii) business competition and (iii) offshoring and lean production. Finally, 91 records were removed, leaving 306 full-length articles in our review portfolio.

## 3. Scientometric Experiments and Analysis

### 3.1. Chronological Publication Trend

**Figure 2:**
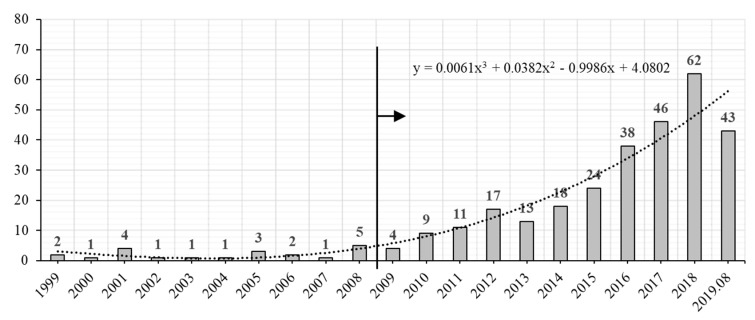
Year profile of indexed documents.

Figure 2 displays the number of papers published annually from 1999–2019 in the portfolio. Obviously, research on G&SL was virtually stagnant until 2009, and since 2010, it has increased significantly year by year. By 2018, a staggering 62 articles were searchable. The vigorous development of academic research indicates the expansion of the scope and branch of G&SL. Furthermore, from the publication number and the recent discussed topics of G&SL, it is evident that the public awareness, market acceptance, social demand and real-world practice of sustainable logistics measures are undergoing remarkable ascent.

### 3.2. Journal Allocation and Co-Citation Analysis

**Figure 3:**
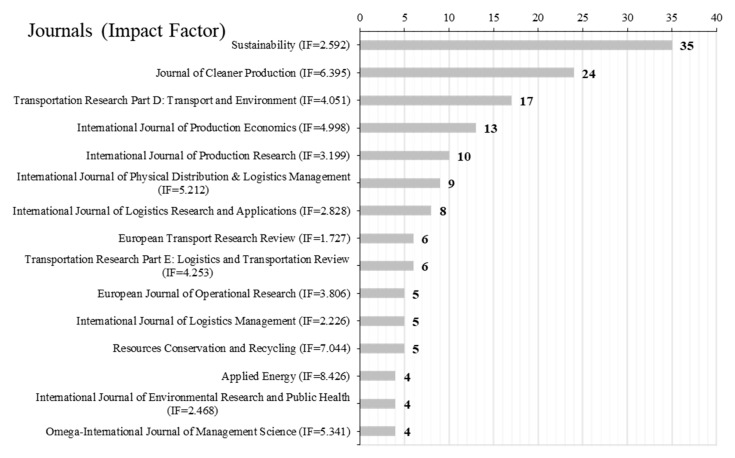
Rank of journals in G&SL publication number.

All 306 documents were found in 81 different journals. As shown in Figure 3, the top 15 journals contributed 155 papers, accounting for 51% of the total. The impact factors of journals were also attached based on the Journal Citation Reports (2018). *Sustainability* ranks first (35, 11.4%), followed by *Journal of Cleaner Production* (24, 7.8%), *Transportation Research Part D: Transport and Environment* (17, 5.6%) and *International Journal of Production Economics* (13, 4.2%). Among the top 15 journals, eight are from UK, four from The Netherlands, two from Switzerland, and one from Germany. The papers are mainly distributed in the three academic fields of environment, traffic engineering and operations management, but they obviously account for a larger proportion in the environmental science and sustainable field, which is in line with the connotation of G&SL.

**Figure 4:**
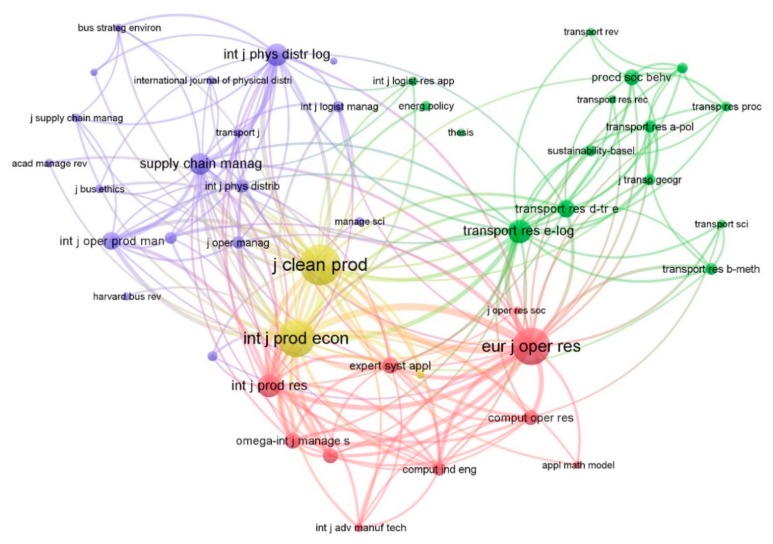
Mapping of the journals co-cited.

As shown in Figure 4, among the 12,408 references (corresponding to 2349 different journals), a network of 46 items and 1025 links was formed by identifying the journals that had been cited more than 50 times. In general, the journals that influenced G&SL research are concentrated in three interrelated clusters. First is the operations research (OR), such as *European Journal of Operational Research* (TLS = 13,076, citation = 494), *International Journal of Production Research* (TLS = 252, citation = 8260), *Expert Systems with Applications* (TLS = 4748, citation = 153), *Omega* (TLS = 5139, citation = 150) and *Computers & Operations Research* (TLS = 4234, citation = 137), which can offer quantitative methods for the decision-making and optimization issues related to G&SL. The second cluster is transportation research (TR), such as *Transportation Research Part A* (TLS = 2057, citation = 103), *Part D* (TLS = 3883, citation = 176), *Part E* (TLS = 7576, citation = 260), and *Journal of Transport Geography* (TLS = 2089, citation = 91), which accumulates enormous knowledge towards transportation planning, technology and operations that can enlighten G&SL research from real-life transport demand and practice. The third cluster, including *Supply Chain Management* (TLS = 6546, citation = 232), *International Journal of Physical Distribution & Logistics Management* (TLS = 6357, citation = 235) and *Journal of Business Logistics* (TLS = 2837, citation = 96), etc., reveals that a large amount of G&SL research was conducted based on the research foundation of logistics and supply chain management (SCM). Among all the publications, *Journal of Cleaner Production* (TLS = 13,799, citation = 555) and *International Journal of Production Economics* (TLS = 13,903, citation = 495) are the two most co-cited journals. They often act as hubs, integrating the results of OR, TR and SCM with social, environment or economic implications to provide cross-domain knowledge crucial to the diverse development of G&SL.

### 3.3. Countries/Organizations Collaboration Analysis

**Figure 5:**
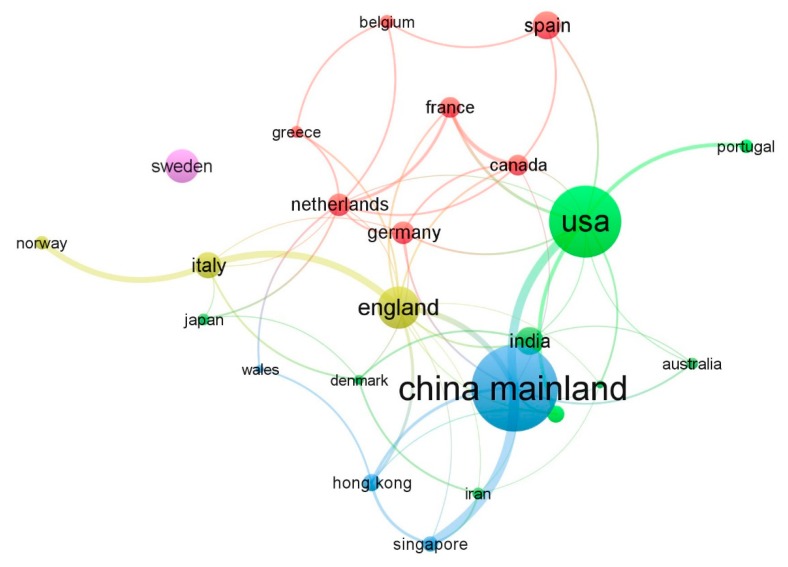
Mapping of countries/regions contribute to G&SL research.

Table 2 lists the countries or regions that are actively studying G&SL, showing six measurements, including number of publications (NP), TLS, average citation year, total citations, average citation per country/region, and average normalized citation. The average normalized citation was calculated by dividing the total number of citations by the average number of citations published per year [34]. Figure 5 displays the collaboration network among countries and regions. The minimum number of documents and citations for a country was set at 5 and 30 respectively. Finally, a map with 25 items and 58 links was generated.

According to Table 2, G&SL research is widely distributed, especially in Europe, Asia, and North America, which is a field of worldwide concern. Mainland China has the most publications, but the United States has the highest total citation. Other countries/regions such as Italy, Singapore, Hong Kong, and Taiwan present a lower number of publications; however, they keep significant figures of average normalized citation which can strongly express their high influence. Besides, most of the documents contributed by these countries/regions were published in the last three years, which means they are playing an increasingly active role in promoting G&SL.

Two evidence can be observed from Figure 5. First, based on a partnership, the global G&SL research is divided into four communities. Therein, two communities are leaded by European counties, such as UK, Spain, The Netherlands, and Italy, while the other two communities are “Mainland China-Hong Kong-Singapore” and “United States-India-Australia-Portugal-Taiwan”, dominated by China and USA, respectively.

Second, the international collaboration is not significant. Taking mainland China for instance, about 70 percent of 49 publications are completed entirely by domestic institutions. The Swedish publications do not have any co-authors from other countries or regions. This phenomenon may be due to the large differences in the background and model of G&SL development in different countries [35]. Moreover, the knowledge gap caused by the wide extension of G&SL and the scattered knowledge structure make the research still focus on the respective fields of researchers, such as sustainable development [36], environment governance [37] and transportation planning [38]. Therefore, at present, the cooperation between academic institutions of different backgrounds has not been widely carried out.

**Figure 6:**
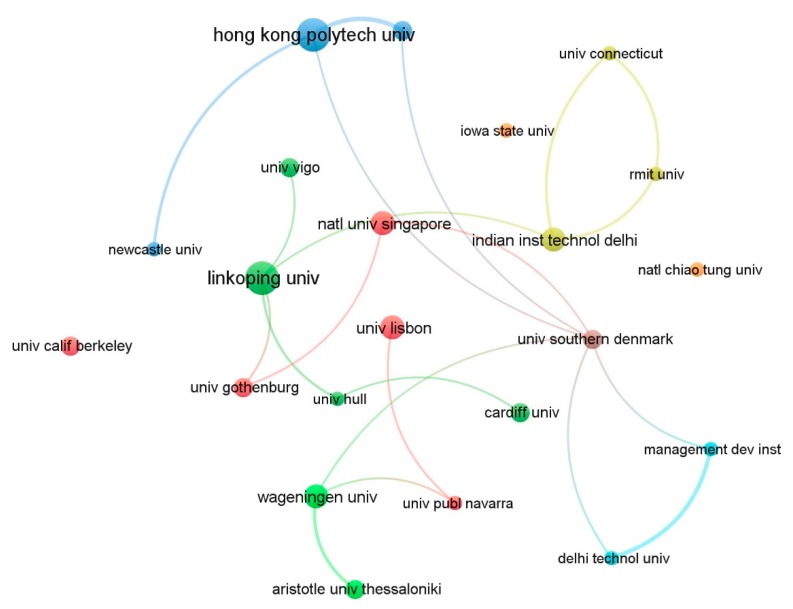
Mapping of global collaboration network among organizations.

Among the 402 organizations that contributed to G&SL research, those with more than five documents and over 30 citations were built into a network of 22 items and 22 links, as shown in Figure 6. None of the organizations published more than 10 papers (3% of 306) and the studies were relatively independent. Therefore, it can be argued that no organization has yet been able to lead G&SL research so far. However, some of the institutions located in Asia Pacific and Europe have a higher reputation in G&SL due to higher citations, including the Hong Kong Polytechnic university (Hong Kong, 388 citations), Wageningen University (The Netherlands, 370 citations), Aristotle University of Thessaloniki (Greece, 324 citations), National Chiao Tung University (Taiwan, 330 citations), Iowa State University (USA, 206 citations), University California Berkeley (USA, 160 citations) and Nanyang Technological University (Singapore, 137 citations). In addition, Figure 6 also shows insufficient collaborative research across organizations.

### 3.4. Influential Research Highlight

**Figure 7:**
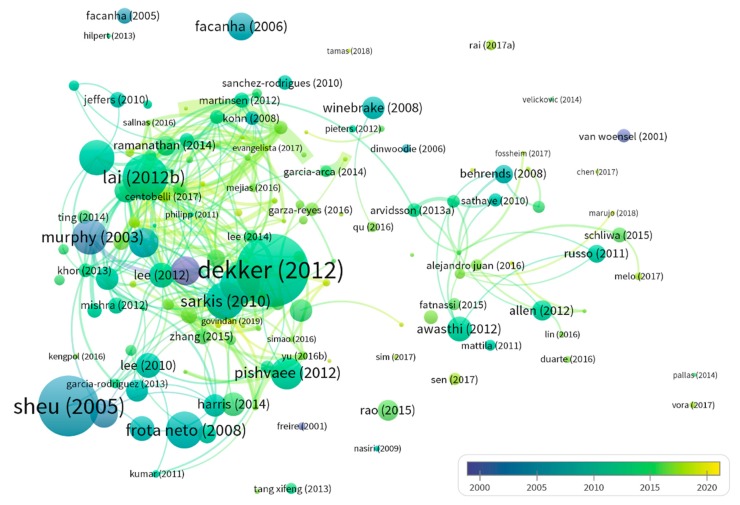
Mapping of the influential documents and their co-citation relationship.

Through the document co-citation test of the portfolio, the most influential G&SL publications in the past two decades were analyzed and the co-citation network was constructed. In VOSviewer, the minimum number of citations was set to 30 to build a co-cited visual network map of 83 items and 350, as shown in Figure 7. The nodes in the map denote the documents that were identified by the first author name and the publication year. The colors of the nodes and the links represent the time of publication and the time of two documents that are co-cited, respectively. The co-occurrence of the literature shows an obvious type of “local concentration and overall dispersion”, indicating that some G&SL studies were widely recognized and produced some common ideas and results. Most papers with high citation appeared around 2010, which was a landmark year for G&SL research. The co-citation time series indicate that G&SL knowledge spreads faster and faster.

The top 15 most cited papers are presented in Table 3, showing their publication year, title, TLS, citation counts and topics. The most cited study was by Dekker et al. [39], one of the first methodological studies to link the operations research knowledge (such as design, planning, and control) to the field of green logistics. The second is Sheu et al. [40], whose main contribution is to propose a modeling technique for sustainable logistics operations and management decisions to maximize supply chain profits. These were followed by papers by Lai and Wong [41] and Ubeda et al. [42], which focused on using the scenario-based approaches, such as the questionnaire and case study, to evaluate the environmental performance of green logistics practices. The main topics of other highlighted documents involve: (i) management insights from industrial practices [43,44]; (ii) multi-criteria evaluation system for green logistics (e.g., policy [45], environment [46], and transportation planning [47]); (iii) network facilities design and optimization [48,49]; (iv) reverse logistics [50,51]; and (v) enterprise responsibility and third-party logistics [52].

### 3.5. Keywords Co-Occurrence Analysis

**Figure 8:**
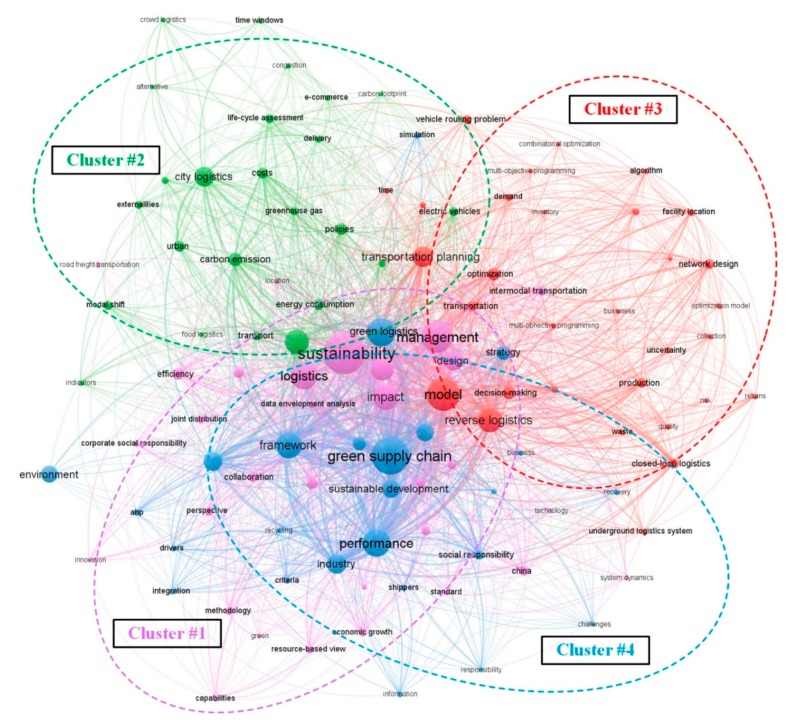
Mapping of co-occurred keywords.

The keywords co-occurrence analysis was conducted to describe the internal composition and structure of G&SL and to reveal the frontiers [31]. The options “All Keywords” and “Full Counting” in VOSviewer analysis were checked to obtain a holistic intellectual landscape of G&SL research. Before the scientometric test, the keywords, such as “third-party logistics providers” versus “3PL”, “transport” versus “transportation”, which are necessary due to differences in expression, were manually simplified on the original data file. The minimum occurrences of each keyword was set to 4, forming a network of 112 nodes representing keywords (1455 keywords in all documents) and 2067 links, as shown in Figure 8.

Figure 8 displays the mainstream of research keywords in G&SL and their co-occurrence relationships. Divide these keywords into four clusters and distinguish them with different colors. Therein, Cluster #1 contains 18 items focusing on the practice and management of logistics sustainability (e.g., collaboration, case study and intermodal transportation), while Cluster #2 covers 25 items, concentrating on the environmental issues of freight transport, such as carbon emission, energy consumption and lifecycle assessment. Cluster #3 (34 items) and Cluster #4 (34 items) emphasize on the “model, planning and optimization” as well as the “supply chain performance, development strategy and competitiveness”, respectively.

Table 4 shows the detailed information of the significant keywords. The top 10 most frequently studied and highly connected terms are sustainability (Feq. = 80, TSL = 547), green supply chain (Feq. = 68, TSL = 629), management (Feq. = 58, TSL = 411), model (Feq. = 55, TSL = 394), green logistics (Feq. = 48, TSL = 325), performance (Feq. = 47, TSL = 367), logistics (Feq. = 46, TSL = 299), framework (Feq. = 43, TSL = 356), impact (Feq. = 41, TSL = 312) and reverse logistics (Feq. = 39, TSL = 323). These keywords play a critical role in forming G&SL research topics and connecting major branches of knowledge. According to the metric of average citations, the following keywords, including transportation, environmental sustainability, production, reverse logistics, and efficiency, aroused a lot of attention.

**Figure 9:**
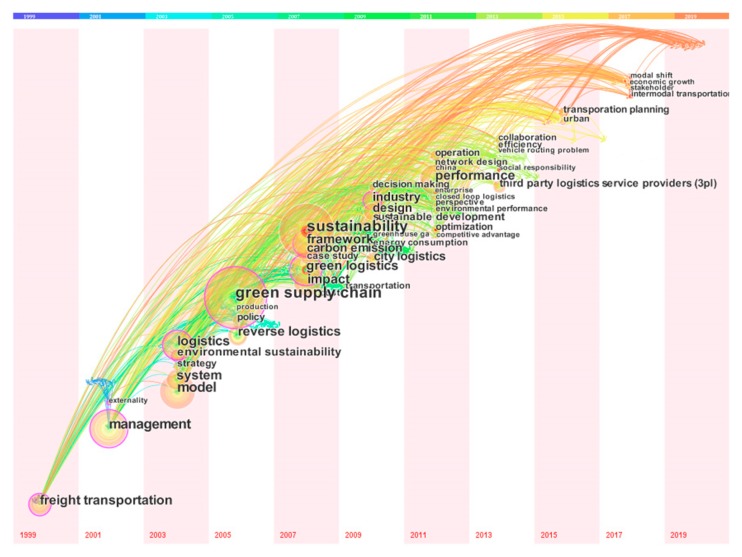
A time zone view of clustered research themes: 1999-2019.

Keyword co-occurrence network is a static expression of a particular area that does not take into account changes over time in the manner that the terms are used [54]. Figure 9 shows a time zone view of keywords that occur more than eight from 1999 to 2019. Each term is arranged in chronological order to present the trend and interaction of keywords. Studies on management, model and green supply chains had been published extensively before 2005 and had been going for a long time, showing that these early topics are still the hotspots of current research. In contrast, articles related to collaboration, transportation planning, modal shift and stakeholder were published from 2015 to 2017, which are emerging themes discussed frequently in recent years and may become the hotspots of future research. Additionally, a large proportion of the keywords were proposed between 2007 and 2015, indicating that G&SL research was greatly enriched during this period. Table 4 presents the time span of all highlighted keywords.

## 4. Discussion

### 4.1. Knowledge Taxonomy of Current Research

**Figure 10:**
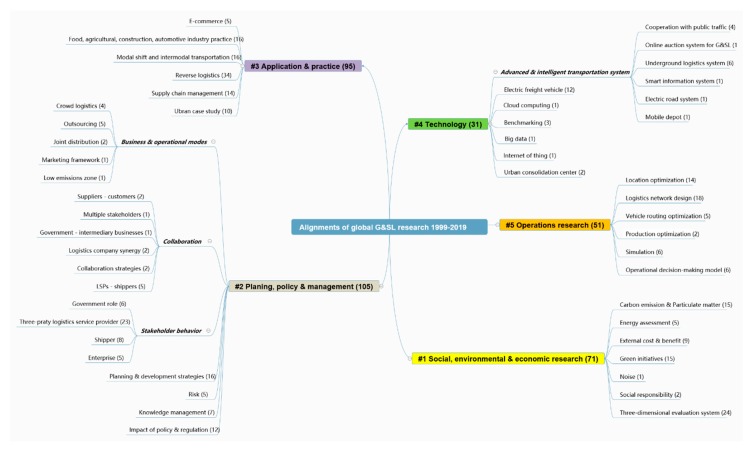
The knowledge taxonomy of G&SL themes.

Through the aforementioned analysis, the research progress, evolutionary trend, and hot-discussed topics of global G&SL are clarified. However, the generic scientometric results cannot accurately reflect the explicit division of the multifarious knowledge of a domain [31]. Based on the clustering analysis of high-frequency keywords, a comprehensive taxonomy of G&SL knowledge from 1999 to 2019 was further proposed, and each separated branch was thematically discussed in-depth subsequently. Topics with similar attributes were integrated into different categories of themes and manually renamed to make the taxonomy more compact and easy to understand. Figure 10 demonstrates the mind mapping of G&SL research themes, where a total of 5 alignments and 50 sub-branches are assembled. The number of representative articles of each theme was also attached.

#### 4.1.1. Evaluation on the Social, Environmental and Economic Impacts of G&SL Initiatives

Nearly a quarter of the literature (71 out of 306 papers) focused on evaluating and quantifying how the potential green logistics initiatives improve the “triple bottom line” (i.e., social, environmental and economic performance, SEE) of existing freight activities. The subjects of these studies were basically originated from four aspects: carbon emission, energy consumption, social sustainability, and external cost-and-benefit. Mattila and Antikainen [15] provided a backcasting method for the long-term prediction of greenhouse gas emissions and fossil fuel consumption in long-distance freight transport, considering the sustainable goals and policies developed by the EU governments. Similar research was conducted for assessing the U.S. scenario [46]. A questionnaire survey conducted by Makan and Heyns [55] found that the pressures from consumer, brand protection, top management, and cost-saving and revenue are the major drivers for freight organizations to implement the sustainable initiatives. Khan et al. [56] modeled the impact of G&SL performance on the countries’ economic development and macro-level social and environmental indicators. Papoutsis et al. [57] and Solomon et al. [58] both maintained that logistics sustainability is closely related to operational efficiency and social acceptance from an economic and environmental perspective. Through the expert scoring, Morana and Gonzalez-Feliu [59] identified the most prominent factors affecting the sustainability of urban logistics are monetary saving, services quality, and customers’ satisfaction rate (economic), pollution emissions and congestions (environmental), and the number of employment created/destroyed (social). Social and environmental activities play a more important role in promoting sustainable logistics than financial-economic activities [60]. Rashidi and Cullinane [61] found that the national logistics industry with high SEE index has the following features: (i) well-planned logistics network infrastructure; (ii) high quality of service operators; (iii) shipments tracing technology; and (iv) efficient timetable scheduling.

Another part of emphasis was given to SEE performance of G&SL based on logistics operations and business. Guo and Ma [62] evaluated the energy consumption and emission level under different logistics business modes, concluding that the third-party logistics provider and the joint distribution modes have obvious environmental advantages in developing green urban distribution. Wang et al. [63] found that green logistics performance would impose positive effects to the exporting countries in the international trade. Herold and Lee [64] investigated the carbon reports disclosed by some giant international logistics enterprises, e.g., UPS, FedX and DHL, and compared their sustainability-related strategies, namely legitimacy-seeking arguments versus energy and emission reduction. In addition, a variety of qualitative analysis measures, such as fuzzy multi-criteria evaluation modeling [65], data envelopment analysis [66], and analytic hierarchy processes [67] were also widely applied to illuminate the logic between SEE performance and G&SL.

Except for the three-dimensional evaluation system, some scholars also analyzed the critical success factors and barriers for G&SL initiative implementation from the SEE perspectives. For instance, Arslan and Sar [68] found that the managers’ intention towards green logistics initiatives is generally determined by the environmental attitude, perceived behavior control and subjective norm. Besides, government subsidizes [69] and internalization of externalities [70,71] were considered to be the effective models to reduce negative external cost in the logistics industry, thus promoting the greening process of the logistics market.

#### 4.1.2. Planning, Policy and Management Research of G&SL

This knowledge branch focuses on two basic G&SL topics, (i) the planning, development, and policymaking from industrial level, and (ii) the collaboration strategy and management from project level. For the former, Lindholm and Blinge [2] indicated that the public support, stakeholder partnership, and excellent management skills are the most significant factors to achieve sustainable development of the logistics industry. The coordination among metropolitan economy, logistics infrastructure investment, and industrial chain upgrading is the essential foundation of G&SL [36]. Integrating freight activities into the general planning procedure or transport planning is also considered important for the implementation of G&SL. Shankar et al. [72] quantified the dynamic uncertainties and intrinsic sustainability risks of freight transport and stated that most of the risks were socially induced rather than financially driven. The risks of multimodal green logistics were analyzed by Kengpol and Tuammee [73]. A system dynamics simulation conducted by Sudarto et al. [74] revealed that the economic performance of G&SL is directly affected by freight policy, while environmental performance is indirectly affected. Klumpp [75] proposed two strategies to develop green logistics, namely encouraging public investment and imposing heavy taxes on carbon raw materials.

For the latter, the collaboration and game among logistics service providers (LSP), government, shippers, and enterprises are paid more attention. Commonly, a positive cooperation strategy of stakeholders will significantly improve the operational performance of G&SL [76] and even the entire supply chain [19]. Therein, the benefits brought by the collaboration between suppliers and customers [77] and LSPs-shippers [78] are particularly salient. The government plays a dominant role in the knowledge dissemination [79] and economic incentive of greenization [20], leading to the innovation of logistics technology. Moreover, the shippers’ willingness to pay for G&SL products [80], the exploitation of green logistics knowledge [81], as well as the gaps between green logistics demand and supply [82] also aroused research attention.

Furthermore, several novel business and operational modes of logistics aiming at improving the sustainability in transportation process were proposed, e.g., freight consolidation [83], smart logistics [22], and low emissions zones [84]. The most hotly debated topics are outsourcing and crowd shipping (CS). CS, proposed for the last-mile delivery problem, is a concept that means the parcels and passengers are co-transported along a passenger trip [85]. According to Ameknassi et al. [86], freight transportation, warehousing, and reverse logistics are the three major outsourced logistics activities. The outsourcing strategy has proven to be advantageous in reducing energy use, global warming, and supply chain risk, compared with common logistics operations [87].

#### 4.1.3. Real-World Application Areas and Practices

Over the past decade, research on the G&SL practices were carried out over a broad range, including SCM, reverse logistics (RL), e-commerce, urban distribution, multimodal transport, and other dedicated logistics such as food [88] and manufacturing [89]. Much valuable experience and instructions can be obtained from real-world applications. For example, the unsustainability of the supply chain is largely due to the poor logistics practices in the downstream [90], which specifically refers to transport operation delay [91], poor communication [91] and the lack of effective management of carbon footprint [92]. A sustainable SCM is an effective measure to improve the competitiveness, financial and environmental performance of logistics enterprises. However, this is not absolute, Hazen et al. [93] believe that some green SCM practices might not necessarily lead to competitive advantage, but make users feel that they are getting low-quality products.

Reverse logistics is convincingly one of the most efficient solutions to reduce environmental pollution and waste of resources by capturing and recovering the values of the used products [94]. Legislation, social image, corporate citizenship, and market competence force enterprises to integrate RL into their supply chains [95]. In real-world application, improving RL sustainability and greening process is the primary goal to optimize the overall supply chain performance. Our review found that most green-related RL studies focused on the network design [96] and system planning [14]. Other topics are waste recycling management [97], benefits assessment [98], reverse operations outsourcing [99] and social responsibility [50].

The unsustainability of urban logistics makes it the most urgent goal of greening. Huge logistics demand, such as rapid business-to-business and business-to-customer logistics activities, make freight transportation in big cities face the dilemma of air pollution, poor accessibility, and livability [31]. The practice of integrating green logistics planning into smart cities construction has been carried out for a long time, especially in Europe, mainly including last-mile delivery [100], traffic management [101] and lean logistics [102].

Compared with G&SL in urban domain, the sustainability issues regarding inter-city or regional logistics are more emphasized on the intermodal application. The shift of road-based modal to other transportation system, such as rail and water has the potentials of ensuring environmental sustainability, flexibility, and cost reduction [17]. However, despite the encouragement by the government, the practice of intermodal transport is still in a preliminary stage due to the difficulties of infrastructure investment [103].

#### 4.1.4. Emerging Technologies Proposed for G&SL Development

Developing advanced facilities and technologies is a sustainable and forward-looking solution to meet the challenge of freight transport. Many emerging logistics systems were proposed in recent years. Such as urban consolidation center [104], electric road system [105], intelligent transportation system [106] and packaging benchmarking system [107], etc. Meanwhile, some soft applied techniques, such as big data [108], internet of things [109] and cloud computing platform [110], have also been applied to logistics operations to support the sustainable development of the emerging systems.

Electric vehicles (EVs) technology, which has been widely applied in passenger transport, is also waving a revolution in the field of G&SL. Current research on freight EVs mostly focuses on energy efficiency [111], fleet optimization [16] and environmental benefits [112]. Simulation results from various cities show that EVs achieve extremely high benefits in carbon emission reduction, with over 80% relief rate tested by Giordano et al. [112].

For reducing the negative externalities such as traffic congestion and disturbance, another interesting concept, i.e., transferring the ground logistics process to underground space, namely the Underground Logistics System (ULS), has aroused increasing attention. ULS refers to using a group of hierarchical underground nodes, pipelines, and tunnels to distribute cargo flows in and between cities with 24-h automated operations [113]. ULS can be designed as a network form connecting urban logistics parks and last-mile delivery, or a dedicated underground container line established between seaports and urban gateways, leading to huge environmental and social benefits (e.g., energy-saving, accidents and congestion mitigation and improving urban logistics capacity, etc.) [114]. So far, the technological feasibility of several ULS projects was acknowledged, yet the large-scale implementation has not started due to the relatively high construction cost and low public awareness [5]. For this reason, the collaborative strategy of retrofitting existing urban rail transit systems, such as trams, light rail or subways, to achieve mixed passenger-and-freight transport has received higher recognition and was successfully stepped into engineering practice in some European cities [115]. Compared with ULS, the collaborative modes are easier to implement, since the dual use of transportation infrastructures would moderate the system cost to an acceptable level [49,116].

#### 4.1.5. Operations Research and Optimization Methods for G&SL Decision-Making

The operations research (OR) of G&SL issues that are originated from real-world applications is always being a well-concerned topic because it is directly related to the quality of some critical decision-making in logistics operation. The OR method applied for G&SL is defined as a better of science to identify the trade-offs between environmental aspects and costs, so that the corresponding decisions such as location, transportation, warehousing, and inventory can be optimized and the limited resources can be reasonably assigned [39]. Dekker et al. [39] classified the application of OR in green logistics as follows: logistics services network design [48], facility location [117], vehicle routing problem [118], inventory management [40] lifecycle production optimization [119], supply chain planning, control, and procurement [120,121] and model choice [122]. A variety of OR techniques, such as heuristic algorithms [121], stochastic programming [53], and robust optimization [123], were developed for the above issues. In addition to the objectives of general logistics planning e.g., cost and efficiency, the G&SL version focus more on the minimization of environmental influence, e.g., carbon emission and energy consumption. Currently, OR is increasingly applied to optimize the G&SLs’ decision-making in a complex scenario set, such as demand uncertainty [48] and facilities failure [124].

### 4.2. Research Gaps and Agenda

Through the above scientometric analysis and thematic discussion, the comprehensive research trend, mainstream academic topics, and knowledge taxonomy of G&SL domain were revealed. Although researchers and practitioners achieved substantial results in promoting G&SL theory and practice, there are still some shortcomings that need to be elaborated in future studies.

#### 4.2.1. Limitations of Global Collaboration and General Evaluation Framework

In terms of research model, international cooperation is still lacking. The broad applicability of most G&SL knowledge based on local cases deserves further discussion, such as planning methods and evaluation systems. European countries made great efforts in rebuilding the integration of green logistics. However, the lack of international cooperation and universal solutions hinders the dissemination and deepening of knowledge, and the current achievements are far from enough to promote the globalization of G&SL, which is reflected in the imbalance of global G&SL practice.

To fill this gap, although it is recognized that logistics policy has a strong regional character, cross-institutional and cross-national collaborative research on market operation, industrial metrics, technology innovation and macro development strategies should be strengthened under the trend of supply chain globalization. For example, more attention can be paid to the horizontal comparison of green logistics mode, scheme and performance under different case backgrounds. Additionally, more empirical studies are needed to be carried out in some developing countries in Asia and elsewhere in the world, considering they are the fast growing economies with higher population and logistics demand.

#### 4.2.2. Complement Research from a Global/Holistic Perspective

Although the knowledge branch of research is flourishing, it is acknowledged that there is still a need to supplement the overall or holistic research to improve the knowledge system of G&SL. Research on sustainability and green has always been complex and multi-variable, interactive, with far-reaching implications. Besides, sustainability and green are public and social issues. Current theoretical applications are limited to the analysis of local or one-way relationships, such as LSP/retailer/carrier responses to green policies, planning and performance evaluation of green and sustainable initiatives.

The operation and decision-making of G&SL involve many stakeholders, such as local authorities, manufactures, LSP, carriers, customers, and even the sharers of transportation resources. The impact of G&SL should also be long-term and dynamic. Thus, the whole picture includes multiple perspectives, such as the dynamic evaluation of the whole life-cycle of green logistics practice, the decision interaction among multiple stakeholders, and the follow-up research and report on a new green technology or practice.

#### 4.2.3. Lack of Effective Platform to Accelerate the Research of Innovation Technology

Without green innovation technologies, the effect of implementing G&SL from a management perspective alone is minimal. However, it takes a lot of time for some innovative technologies that can fundamentally improve the negative effects of logistics to move from laboratory to application. Applications such as the EV took decades to implement [125]. Although the technology is constantly updated and improved, more management lag. Another competent concept, the ULS, ASCE has published a feasible technical system as early as 1998 [126], but only in a few countries has it been publicly piloted in recent years.

The introduction of a new thing does require a long period of demonstration, such as the reliability of the technology, the acceptability of the market and the ambiguity of the real benefits. However, the problem is often the gap and lag in the research of application management in the transition from technical problems to market application and practice management. Therefore, building effective platforms based on multidisciplinary, cross-organizational collaboration to accelerate the research and application of innovative technologies is particularly important for G&SL practices, such as ULS, RL, and CS. Such calls are all the more urgent in their own research.

## 5. Conclusions

The concept of green and sustainable logistics has received increasing attention and consideration government sectors, scholars, practitioners, and international organizations. A large amount of practical achievement was made at both the industrial and theoretical levels. This study reviewed 306 valuable contributions regarding G&LS over the past two decades through a three-step review program. They were described in year publication, journal allocation and citation counts. Then, the bibliographic networks of countries, organizations, journal and document co-citations, keyword co-occurrence and timezone clusters of research themes were visualized to help understand the overall research status and academic progress worldwide. Grounded in the scientometric analysis, an integrated knowledge taxonomy of the G&SL field was presented, including five major alignments and 50 sub-branches.

Results indicate that the chronological publication of G&SL shows a trend of rapid increase. The quantity of literature published in 2018 is fifteen times more than that of 10 years ago. *Sustainability*, *Journal of Cleaner Production*, *Transportation Research Part D: Transport Environment* and *International Journal of Production Economy* are the top four journals, which contributed over a quarter of all G&SL papers since 1999. The maps of journal allocation and co-cited journals show that the current research is most relevant to the environmental science and transportation science. In terms of countries, China, the United States, the UK, Sweden, and India are the major territories of G&SL research. The network across co-authored organizations and countries revealed that the collaboration among different research communities is not strong. Hence an active and robust global collaboration atmosphere has not formed yet.

The map of co-occurred keywords showed that the most frequently discussed G&SL themes in each cluster were sustainability and management (cluster #1), freight transportation and carbon emission (cluster #2), model and reverse logistics (cluster #3), and green supply chain and green logistics (cluster #4). The timezone view of keywords showed that articles related to collaboration, transportation planning, modal shift and stakeholder were largely published during the recent years. On this basis, the knowledge taxonomy of G&SL was manually synthesized from five aspects: (i) evaluation on SEE impacts of G&SL initiatives; (ii) planning, policy, and management research; (iii) real-world application areas and practices; (iv) emerging technologies and (v) operations research and optimization methods for G&SL decision-making.

Finally, the potential roadmap for filling current research gaps was recommended, which were divided into three streams: (i) more global research collaboration should be advocated to jointly develop and supplement the comprehensive evaluation framework of G&SL performance; (ii) future research efforts could focus on the interactive and dynamic relationships among sustainable development goals, green policies and the decision-making of multiple stakeholders; (iii) the application-oriented platforms and management research for some most advanced green logistics initiatives would be highly beneficial in promoting G&SL innovation.

However, it should be noted that the data used in this study was confined to those research articles and review articles that were published in the peer-reviewed journals, and they were retrieved only from the two mainstream databases considering the applicability of software. Although the indexed documents could represent most of the convictive viewpoints of G&SL research, some valuable articles that were published in other forms or included in other databases might be overlooked inevitably. To sum up, this review has great room for improvement in terms of material selection. A systematic investigation incorporating valuable conference proceedings, reports, and books in the field of green logistics or green supply chain is expected to portray a more comprehensive knowledge map for future research. Additionally, the in-depth review of the hotspot themes in G&SL domain e.g., OR application and SCM, may also contribute to multidisciplinary integration and interaction.

## Figures and Tables

**Table 1:** Results of literature retrieval and selection.

Databases	Web of Science Core Scopus	Initial Records: 418
		Initial Records: 742
Logical statement	TI = ((“sustainable” OR “green” OR “sustainability” OR “environmental” OR “ecofriendly” OR “ecological”) AND (“logistics” OR (“reverse” AND “logistics”) OR ((“freight” OR “goods” OR “cargo”) AND (“transport” OR “transportation” OR “delivery” OR “distribution” OR “movement” OR “shipment” OR “supply”))) OR (“electric” AND (“truck” OR (“freight” AND “vehicle”)))) AND Language: (English) AND Type: (Article) AND Time span: (1999–2019)	Valid records (first-round filter): 397
Inclusion criteria	(i) green logistics initiatives and practices; (ii) strategy, policy, environmental evaluation, review and technology; (iii) planning and operational research, etc.	Final records (second-round filter): 306
Exclusion criteria	(i) non-peer-reviewed journals; (ii) lack of references, authorships or full text; (iii) less than 5 pages; (iv) Articles do not relate to G&SL (e.g., generalized supply chain management, lean production, market and purchasing, and public transport)	

**Table 2:** Summaries of countries/regions active in G&LS research.

Country/Region	Territory	NP	TLS	Ave. Year	Total Citations	Ave. Citation	Ave. Norm. Citation
China Mainland	Asia	49	36	2017	234	4.78	0.57
United States	North America	41	30	2012	1388	33.85	1.28
England	Europa	24	28	2014	488	20.33	1.16
Sweden	Europa	19	0	2014	300	15.79	0.88
India	Asia	16	6	2018	90	5.63	1.07
Spain	Europa	16	6	2014	355	22.19	0.86
Italy	Europa	15	18	2017	284	18.93	1.68
The Netherlands	Europa	13	18	2011	524	40.31	1.33
Germany	Europa	13	10	2014	116	8.92	0.71
Canada	North America	12	14	2014	285	23.75	0.98
France	Europa	12	12	2014	203	16.92	0.86
Hong Kong	Asia	10	12	2018	394	39.4	1.53
Taiwan	Asia	10	8	2017	456	45.6	1.47
Singapore	Asia	9	16	2017	214	23.78	1.64
Belgium	Europa	8	6	2014	152	19	1.18
Portugal	Europa	8	4	2013	112	14	1.09
Greece	Europa	8	6	2015	326	46.57	0.92

**Table 3:** List of publications with the highest impact in G&SL.

Document	Year	Title	TLS	Citation	Topic Related to G&SL
Dekker et al. [39]	2012	Operations research for green logistics - An overview of aspects, issues, contributions, and challenges	100	330	Operations research
Sheu et al. [40]	2005	An integrated logistics operational model for green supply chain management	20	260	Operations research
Lai and Wong [41]	2012	Green logistics management and performance: Some empirical evidence from Chinese manufacturing exporters	82	167	Management practices
Ubeda et al. [42]	2011	Green logistics at Eroski: A case study	52	146	Management practices
Sarkis et al. [50]	2010	Reverse logistics and social sustainability	104	128	Reverse logistics
Frota Neto et al. [11]	2008	Designing and evaluating sustainable logistics networks	22	128	Operations research
Murphy and Poist [34]	2003	Green perspectives and practices: a “comparative logistics” study	78	118	Management practices
Lin and Ho [45]	2011	Determinants of green practice adoption for logistics companies in China	48	115	Systematic evaluation
Pishvaeee et al. [48]	2012	Credibility-based fuzzy mathematical programming model for green logistics design under uncertainty	24	114	Operations research
Presley et al. [51]	2007	A strategic sustainability justification methodology for organizational decisions: a reverse logistics illustration	46	91	Reverse logistics
Murphy and Poist [44]	2000	Green logistics strategies: An analysis of usage patterns	62	90	Management practices
Lieb and Lieb [52]	2010	Environmental sustainability in the third-party logistics (3PL) industry	0	87	Environmental impact
Hovath [46]	2006	Environmental assessment of freight transportation in the US	12	83	Environmental impact
Awathi et al. [47]	2012	A hybrid approach integrating Affinity Diagram, AHP and fuzzy TOPSIS for sustainable city logistics planning	18	74	Systematic evaluation
Lee et al. [53]	2010	The design of sustainable logistics network under uncertainty	24	73	Operations research

**Table 4:** Summaries of significant keywords and theme clusters of G&SL research.

Cluster ID	Keywords	Occurrence	TLS	Ave. Citation	Ave. Norm. Citation	Time Span
Cluster #1 (purple) Size = 335	Sustainability	80	547	13.9	1.2	2007–2019
	Management	58	411	16.6	0.9	2001–2019
	Impact	41	312	18.4	1.1	2008–2019
	Logistics	46	299	19.1	1.1	2003–2019
	Systems	37	260	19.3	1.2	2004–2019
	Case study	14	108	29.6	1.1	2008–2019
	Efficiency	14	108	32.7	1.3	2013–2019
	China	12	88	27.9	0.8	2011–2019
	Intermodal transportation	12	88	5.2	0.6	2017–2019
	Collaboration	11	73	12.7	0.8	2013–2019
	Stakeholder	10	71	8.2	1.2	2017–2019
Cluster #2 (green) Size = 169	Freight transportation	38	223	15.3	1.1	1999–2019
	Carbon emission	31	197	13.8	1.1	2007–2019
	City logistics	29	118	12.7	1.1	2010–2019
	Policies	14	94	24.5	1.1	2005–2019
	Costs	13	92	11.7	0.7	2008–2019
	Energy consumption	13	72	8.7	0.7	2009–2019
	Electric vehicles	11	74	16	1.4	2015–2019
	Lifecycle assessment	10	68	22.2	1.6	2017–2019
	Modal shift	10	54	6.6	0.8	2017–2019
Cluster #3 (red) Size = 202	Model	55	394	24.2	1.2	2004–2019
	Reverse logistics	39	323	35.7	1.4	2004–2019
	Transportation planning	17	125	6.1	1.4	2015–2019
	Decision-making	16	132	19.5	1.6	2009–2019
	Optimization	16	122	24.9	1.1	2012–2019
	Closed-loop logistics	12	118	14	1.2	2011–2019
	Network design	12	94	27.1	0.9	2011–2019
	Production	12	75	37.5	0.7	2005–2019
	Transportation	12	106	52.1	1.8	2008–2019
	Vehicle routing problem	11	74	31.1	1.5	2023–2019
Cluster #4 (blue) Size = 422	Green supply chain	68	629	23.4	1.1	2005–2019
	Green logistics	48	325	21.9	0.9	2008–2019
	Performance	47	367	17.7	0.9	2011–2019
	Framework	43	356	18.9	1.1	2007–2019
	Industry	29	260	20.4	1.1	2009–2019
	Third-party logistics service providers (3pl)	27	206	11.5	1.5	2013–2019
	Environmental sustainability	26	189	42	1.5	2003–2019
	Sustainable development	26	191	15.6	1.1	2010–2019
	Environment	24	21	15.7	0.7	2009–2019
	Strategy	20	139	23.1	1.3	2004–2019
	Operations	17	137	12.6	0.8	2011–2019
	Urban	13	65	8.1	1.2	2015–2019
	Environmental performance	12	93	27.5	1.3	2012–2019
	Competitive advantage	11	88	30.5	1.3	2011–2019
	Social responsibility	11	106	26.3	1.6	2013–2019
